# From Distance Learning to Integrated Digital Learning: A Fuzzy Cognitive Analysis Focused on Engagement, Motivation, and Participation During COVID-19 Pandemic

**DOI:** 10.1007/s10758-021-09571-w

**Published:** 2021-10-04

**Authors:** Roberto Capone, Mario Lepore

**Affiliations:** 1 Dip. Matematica, University of Salerno, Fisciano, SA Italy; 2 Consorzio di Ricerca Sistemi ad Agenti CORISA, University of Salerno, Fisciano, SA Italy

**Keywords:** Distance learning, Virtual environment, Fuzzy cognitive map, Motivation, Participation, Engagement

## Abstract

This work focuses on Distance Learning during the COVID-19 pandemic to improve students’ motivation, participation, and engagement, trying to contain the drop-out phenomenon. Distance Learning at the time of COVID-19 is an educational methodology and it can be considered the only occasion to keep an educational connection between students and teachers. Experimentation in a Mathematics STEM class was carried out evaluating the impact of Distance Learning on students’ levels of motivation, participation, and engagement, computed through a Fuzzy Cognitive Map. Specifically, it was performed on some affective and interaction parameters derived from using an adaptive e-learning platform and from the answers of a semi-structured questionnaire. The results, which have been analysed through Technological Pedagogical Content Knowledge and Instrumental Genesis theories, show on one hand that Distance Learning is valid as an additional and support methodology but, on the other hand, they highlight the ineffectiveness of completely remote teaching. Therefore, a teaching method that integrates moments of distance teaching with activities carried out in the presence, in the classroom, or in other university environments, is hoped to be used as soon as the emergency is over: a mix of styles, a fluid flow of knowledge between the physical classroom and the virtual classroom. We will call this Integrated Digital Learning.

## Introduction and Motivations

Due to the COVID-19 pandemic, the school has been overwhelmed by rapid changes that have required, in a very short time, to push didactic beyond the traditional methodologies and to open teachers and students to a new reality that has forced them to virtual classrooms. Students and teachers no longer share the same physical space, and they need a new narrative to communicate (Baudrillard 1992). Schools and universities have been closed in many countries. Conferences have been canceled or postponed. Furthermore, scientific determinism does not seem to give the correct answers to the problem’s solution, as there is a lack of absolute measures to contain the pandemic.

Distance Learning (DL), at the time of COVID-19, is, therefore, a didactic of “being there”, as defined by the philosopher Heidegger, in the etymological sense of “being-out” (ex-sisto), being is meant as the projection to the future, an opening to possibilistic horizons. In general, DL was characterized by the use of methodologies and technological tools to keep students’ levels of participation, engagement, and motivation high. In fact, a low level of these parameters can lead to an early drop-out of the course (Jacobsen 2019), whose increment has always been feared (Levy 2007). The drop-out phenomenon is common to university courses held in presence (Roberts et al. 2017) or blended mode, or with the help of e-learning platforms. In the context of the emergency of COVID-19, students have experienced moments of strong emotional stress, with the risk of generating a state of frustration and discouraging them from studying. It was not just a question of using technologies in teaching but of an actual adaptive process to the exclusive use of technologies as the only way to teach. Rabardel’s ideas on instrumentalization and instrumentation (Rabardel 2005) form the theoretical framework for the experimentation. They are integrated into the Technological Pedagogical Content Knowledge Framework (TPCK), showing how the didactic contents delivered in the presence have taken on a new aspect with distance learning (Mishra and Koehler 2007). The didactic contents have not been substantially modified, but there was a need to review some pedagogical and methodological aspects. Some theories on motivation (Skinner 1935; Lepper 1988; Fredricks et al. 2004) were used to analyze the students’ motivation to follow lessons at a distance, to be virtually present in classes (Shonfeld and Magen-Nagar 2020), and to use the teaching tools made available by the teachers (Anthony et al. 2020).

Our research is motivated firstly by the need to analyze how the student’s status, defined in terms of engagement, motivation and participation, could change in an emergency context, secondly, by how to maintain these levels high not only by transferring the teaching contents from face-to-face teaching to completely remote teaching but adapting them according to appropriate teaching models and techniques. Our contribution described in this paper, original concerning what is present in the literature, is to have modeled teaching in an adaptive way using adequate technological tools to cope with panic and frustration in which students suddenly found themselves, trying to stem a possible wide phenomenon of drop-out. A case study was conducted to evaluate whether our didactic models adapted to this emergency situation have been helpful in maintaining a sufficient level of engagement, motivation and participation of the students.

The study was carried out with first-year engineering students of the academic year 2019–2020 in mathematics class. The students regularly attended the lessons of the first semester in presence; the lessons of the second semester, after a first week in presence, took place completely at distance due to the lockdown for the COVID-19 emergency. The case study has nomothetic and ideographic intentions by collecting quantitative and qualitative data. The quantitative data include the interactions of the students on the used e-learning platform, the results of the tests, and a semi-structured questionnaire according to the Likert scale (Likert 1932). In addition, the questionnaire anonymously proposed to students also included open-ended questions from which to infer, through qualitative analysis, the motivational state of the student. Finally, from the qualitative and quantitative data that emerged, a Fuzzy Cognitive Map (FCM) was built to derive the levels of the students’ engagement, motivation, and participation parameters. Based on completely remote teaching, the obtained results are compared with those obtained in a previous experiment (Capone and Lepore 2020) in which the course was conducted in a blended mode in the academic year 2018–2019. The data collected seem to show that, thanks to the use of an adaptive e-learning platform and restructuring of educational content by using technologies, the students have maintained an adequate level of engagement and motivation parameters comparable to those of previous years. On the other hand, the level of participation was lower than the previous experience due to a drop in concentration and frustration of being at the computer the whole day in order to follow the online lectures. The dropout feared at the beginning of distance learning was instead contained within acceptable values. Furthermore, in terms of achievement of competencies, the results achieved by students are comparable to previous years (Capone et al. 2021).

The viral use of distance learning as the only tool to deliver university courses has generated many doubts and questions:

- What didactic strategies must the teacher adopt to ensure that students acquire the same skills as in face-to-face teaching?
- How can students’ interests be kept alive? How to stimulate them to follow the lessons at a distance?
- Is it possible to structure a virtual learning environment in order to get the most out of an adaptive e-learning system?
- Which aspects of face-to-face teaching are compromised with distance learning?

These and many other questions have gripped the teachers who found themselves delivering university courses completely at a distance during the Covid-19 pandemic. This study tries to answer some of these questions by structuring the virtual learning environment for university students in order to get the most out of an adaptive e-learning system with the possibility of formally analyzing the current situation of the student, defined by indices of engagement, motivation, and participation, calculated through an FCM. Starting from this analysis, it is possible to generate personalized feedback for each student that guides him in his online learning path (D’Aniello et al. 2020b; Chang et al. 2020; Lepore and Petruzziello 2021). With respect to completely remote teaching, the first concern of the teacher was to maintain a high degree of student involvement in terms of engagement, motivation, participation because they are considered important elements for the acquisition of skills and because they are directly linked to dropout phenomenon. From these premises, the following research questions arise:

1. How do engagement, motivation, participation change with completely remote teaching?
2. Can an adaptive e-learning system and personalized teaching contribute to effective teaching, even if completely remote, in terms of competencies acquired by students?

The rest of the manuscript is organized as follows. Section 2 provides a literature review regarding the state-of-the-art approaches to improve students’ motivation, participation and engagement. Section 3 describes the conceptual framework to which the experimentation conducted refers, while Sect. 4 describes an embedded case study with both ideographic and nomothetic intentions. In Sect. 5, data from a qualitative analysis and quantitative analysis are reported. Lastly, Sect. 6 concludes the work with final remarks and future works.

## Related Works

In the literature, there are many references to the benefits of using technologies in teaching. Some authors refer to the role of motivation and engagement (Kong et al. 2003; Xiong et al. 2015), others to the increase of participation (Wolcott 2003; Li et al. 2020), all elements considered important to foster a student-centered learning environment. Some argue that a high level of these parameters can stem the dropout phenomenon (Hidalgo and Abril 2020; Jacobsen 2019). In fact, it is not uncommon for many students to abandon their studies due to the first obstacles encountered in basic courses such as that of mathematics, especially STEM (Science, Technology, Engineering and Mathematics) students who consider the mathematics exam as a sacrifice to be expiated in order to proceed in their studies (Branchetti et al., 2017). These studies are generally helpful for blended teaching, where some technologies are used in traditional courses. While referring to this extensive literature, this research analyzes a completely new situation, that of university teaching delivered completely at a distance due to an emergency situation that has generated an alteration in the emotional state of teachers and students. The motivation to learn can be defined as a need, a drive supported by expectations, goals, and emotions. A classic distinction is that intrinsic and extrinsic motivation adds a social motivation that intersects with the two (Lepper 1988). Intrinsic motivation means that the student takes a new course just for its pleasure, because it is considered rewarding and motivating in itself. Extrinsic motivation means that the learning activity is carried out for external activities, such as receiving recognition, a certificate, a good grade or avoiding negative situations such as a reprimand. Social motivation leads the learner to take part in activities only to meet new people with similar interests or to do activities with a friend, even if there is no interest in the activity itself.

Student engagement is defined as the time and energy students devote to educationally sound activities inside and outside of the classroom and the policies and practices that institutions use to induce students to take part in these activities (Kuh 2003; Capone et al. 2017). The activities include following lessons, completing the assignment, following the teacher. However, recently the term is used to describe the the learner’s involvement in the whole learning environment (lessons, quiz, assignment, social tools, messaging, forum, etc.). The engagement can be active (learner actively participates in the learning environment by publishing posts in the forum, asking questions, etc.); passive (the learner only answers or vote the posts of other users; she follows the lessons but without asking questions, etc.); disengagement, when the learner has poor participation and interest in the course (Ramesh et al. 2013). The engagement definition of Fredricks et al. (2004) encompasses three kinds of engagement: behavioral, cognitive, and emotional engagement. Behavioral engagement refers to involvement in learning tasks and environments such as time-on-task and attendance; cognitive engagement refers to psychological investment in the process of learning such as the use of learning strategies; and emotional engagement refers to affective reactions to learning tasks and environments such as emotions (Fredricks et al. 2004). The multi-component approach to considering engagement as a meta-construct can be conceptually and practically helpful in researching and developing interventions to improve student engagement (Fredricks et al. 2004). Such an approach can broaden the understanding of engagement (Finn and Zimmer 2012; Fredricks et al. 2004; Lawson and Lawson 2013). For example, if students’ emotional experience is examined along with their off-task behaviors such as disrupting a peer (Skinner et al. 2008), one could better understand how to improve their engagement by providing relevant support for negative emotions such as boredom. Kim et al. (2015) define engagement as cognitive and affective participation in learning activities. They included only cognitive engagement (i.e., using shallow and deep cognitive strategies; Pintrich et al. 1993) and emotional engagement (i.e., experiencing boredom, anxiety, enjoyment, anger, shame, pride, and hopelessness; Goetz et al. 2005) in their definition. They recognize that behavioral engagement is critical. However, in asynchronous online education, there are no face-to-face or synchronous virtual classes to attend and thus, the notion of behavioral engagement is not conceptually clear. For example, students’ login time does not necessarily mean how many hours they studied. They may log in just to download course materials. In addition, although Fredricks et al. (2004) view of engagement as a meta-construct was applied, in the present study, we preferred to keep separated motivation and engagement; in fact, engagement does not occur without the desire to engage, but engagement and motivation are the same, as stated by Martin (Martin 2012). The motivation is strictly related to engagement (Xiong et al. 2015). The motivation can predict the learner’s engagement, while the engagement can predict the learner’s retention in the course.

Another important factor for students’ educational success is participation. Participation refers to the action of taking part in activities and projects, the act of sharing in the activities of a group. The process of participation fosters mutual learning. Collaboration is a helpful tool used within the participatory culture as a desired educational outcome (Dominguez 2012). Participation is also linked to an individual’s emotional issues. Participation is fundamental to engage students both in presence and in online courses. In fact, according to some studies (Harandi 2015; Stanford-Bowers 2008), participation is related to improving student performance. In fact, participation is one of the most important aspects of student learning. When students share their ideas through educational dialogue, they learn to relate so that others can understand the concept expressed. When they ask questions, they learn how to get information to improve their understanding of a topic. Participation is also a valuable learning tool for teachers. Through the students’ questions and the analysis of their feelings, it is possible to highlight what they do not understand and then adapt the teaching methodologies accordingly. Therefore, our idea of participation is in agreement with several works available in the literature: for example, Bergmark and Westman (2018) and Masika and Jones (2016) highlight that when students are participatory and involved in the class, they also have a strong impact on the planning of educational activities. From this also comes a more significant emotional involvement and an increase in social activities that lead students to feel part of a real educational community.

These definitions suggest that engagement extends beyond participation and includes students’ interactions, assignments and forum activities. Even when students are participating and behaving, a teacher cannot assume motivation behind that participation. Teacher, who better understands the construct of engagement, makes an important step toward creating a valid measure of student engagement. The teacher’s presence in discussions can facilitate learning and clarify any questions of learners (Instructor participation in class discussion). Therefore, attention to the discussion especially in the conceptualizing, is very important in learning processes (Capone et al. 2018). For example, in mathematics, it is justified every time the constitution of a mathematician is involved, from the outside (real-world mathematization) or the inside of Mathematics (theory of functions). The approach to the discussion is understood as an attempt to provide a set of tools for analysis and planning by the experienced teacher without reducing the responsibility of the pupils. This Vygotskijan process refers to interactions between subjects (teachers and pupils) who play different roles that must be preserved and valued both in the teaching-learning activity. Also, Sarder supports the importance of establishing an online presence, i.e., the importance of the teacher’s presence to stimulate and facilitate learning by intervening when the learner needs it (Sarder 2014). Kearsley and Shneiderman (1998) proposed the Engagement Theory: A Framework for Technology-Based Teaching and Learning. They observed that “the role of technology in theory is to facilitate all aspects of engagement. The use of e-mail, online conferencing, Web databases, groupware, and audio/videoconferencing significantly increases the extent and ease of interaction among all participants, as well as access to information. The vast array of software tools available for analysis, design, planning, problem-solving, and making presentations enable students to do sophisticated and complex tasks. Technology provides an electronic learning environment that fosters the kind of creativity and communication needed to nourish engagement. They believe that engagement theory represents a new paradigm for learning and teaching in the information age, emphasizing the positive role that technology can play in human interaction and evolution”.

Some authors (Biggs 2011; Biggs and Tang 2010) sought solutions to overcome the typically transmissive traditional teaching. They have proposed a constructive alignment in which the focus is not only on what the teacher is going to teach but mainly on what the outcome of that teaching is intended to be, expressed as the Intended Learning Outcome.

In mathematics education, there are many research experiments to analyze the use of technology for mathematics teaching and learning. (Heid 2005; Faggiano et al. 2017).

Kaput and Thompson (1994), used the metaphor of deep-water ocean waves to illustrate the complex interactions between technology and research in mathematics education, and to us, it seems very suitable. At the surface level are the waves themselves-short term events very much affected by local conditions such as winds and eddies. Then there are swells, of longer duration, of more distant origin, and affected larger-scale local conditions such as temperature and currents. To distinguish waves from swells requires us to analyze wave behavior over more extended periods and to situate that behavior within a larger context of interacting forces. Finally, there are tides whose origins are to be found the frames of reference for swells and waves and whose behavior is measured in time units order of magnitude greater than the others. One can focus on any level of wave activity in isolation from the others, describing its behavior and its effects on craft or beaches. But the different levels of activity interact in subtle yet significant. The Covid pandemic has overwhelmed us like a real storm forcing us to exploit all the potential of technologies to stay connected and carry on the didactics.

More recently, a systematic analysis of 139 recent, published studies of technology interventions in mathematics education, selected from more than 2000 potential studies, has been undertaken by Bray and Tangney (2016). A system of classification, developed as a part of this research, is used to categorize the digital tools, the pedagogical foundations and goals of the activities, and the levels of technology integration in the studies. About full online courses, De Freitas et al. (2015) highlighted that massive open online courses (MOOCs) have been the subject of much polarized debate around their potential to transform higher education in terms of opening access. Although MOOCs had been attracting large learner cohorts, concerns have emerged from the early evidence base centered on quality in learning and teaching provision. There is clear evidence that impressive headline figures on MOOC enrollments often contrast with extremely low course completion rates. Also, motivation and participation are related to the use of technology in learning and teaching Mathematics (Giesbers et al. 2013). This study is part of a line of research already undertaken by the authors and concerns the experimentation of teaching methodologies in STEM courses to motivate students to study mathematics and facilitate their learning. In recent years, several experiments have been conducted by the authors to improve the effectiveness of the teaching action. This is in agreement with several studies in education and mathematics education about effectiveness of information and communication technology, (for example, De Witte and Rogge 2014; Ghavifekr and Rosdy 2015).

In particular, in the academic year 2016/2017, a blended teaching model was tested with half-flipped teaching in the SCALE-UP learning environment (Branchetti et al. 2018). In the academic year 2017/2018, blended teaching was tested using the Just in Time Teaching and Peer-Led Team Learning methodologies integrated with the use of a social platform. In the academic year 2018/2019, the experimentation went on using Augmented Reality to address some crucial topics of the Mathematics course and evaluating student interaction and participation with Fuzzy Cognitive Maps as a systemic structure model for analyzing critical success factors of the learning system (Capone and Lepore 2020).

## Conceptual Framework

This section describes the conceptual framework to which the experimentation conducted refers. Specifically, Sect. 3.1 describes the framework of the Technological Pedagogical Content Knowledge (TPCK) which has helped to interpret the aspects of the Technological Knowledge that have intersected with the pedagogical knowledge and teaching methodologies that the teachers used in distance learning. Section 3.2 describes the idea of instrumental genesis proposed by Verillon and Rabardel (1995) which helped to interpret how technological artifacts have been helpful for triggering good teaching practices aimed at learning. In Sect. 3.3 refers to the theory of Fuzzy Cognitive Map as a tool to describe and model complex systems/environments in a symbolic way, highlighting events, processes, and states.

### Technological Pedagogical Content Knowledge Framework

**Fig. 1:**
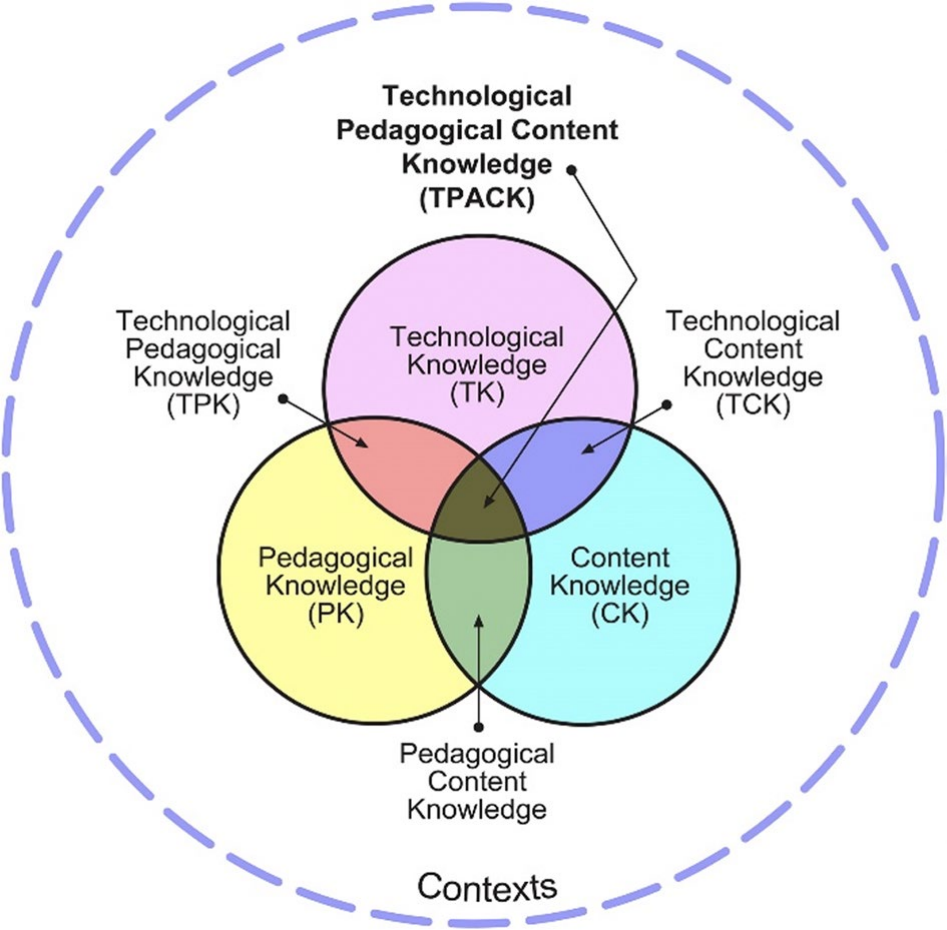
TPACK venn diagram

In this paper, reference was made to the Technological Pedagogical Content Knowledge (TPCK) framework conceived by Shulman in 1986 to define which elements can characterize teaching supported by technologies without neglecting the pedagogical aspects and the specific teaching contents of the discipline. Pierson (1999) illustrated the TPCK model as the intersection of three sets representing the three domains of knowledge. Figure X shows how the acronym TPCK arises from the intersection of three knowledge domains: Technological Knowledge (TK), Pedagogical Knowledge (PK), and Content Knowledge (CK). Gess-Newsome (1999) speaks of two different models: the integrative and the transformative one; in analogy with chemistry, the integrative model can be seen as a mixture of elements, while the transformative model can be seen as a compound. In the integrative model, the didactic contents of the discipline, the pedagogical aspects, and the technology constitute three separate domains that are merged into a single element during the classroom lesson. In the transformative model, the disciplinary contents are combined with pedagogical elements and with technology in a holistic way providing greater teaching support than the simple combination of the parts. Mishra and Koehler (2006) make further clarifications by describing the meaning of the intersections between TK and CK, between PK and CK and between TK and PK and subsequently from their intersection they explain the meaning of the intersection of the Technology, Pedagogy, Content, and Knowledge elements, such as schematically shown in Fig. 1.

They specify the following: “Pedagogical content knowledge (PCK) is concerned with the structure, organization, management, and teaching strategies for how the particular subject matter is taught. Technological content knowledge (TCK) is related to how one specific subject matter is represented in technology-rich environments. Teaching with technology requires knowing not only the subject but also how the subject matter can be changed with the application of technology, and this knowledge is called TCK. Technological pedagogical knowledge (TPK) is concerned with how teaching and learning change as a result due to integrating technology into instruction and how a teacher should be able to choose a particular tool for one specific task considering its affordances and limitations. Technological Pedagogical Content Knowledge (TPCK) “is an emergent form of knowledge that goes beyond all three components” (p. 1028). According to the transformative model, TPCK is different from “knowledge of a disciplinary or technology expert and also from the general pedagogical knowledge shared by teachers across disciplines” (p. 1029). This template helps to read some of the results of this research. In particular, it helps to interpret the attitude of teachers in the face of innovation in their didactic. In fact, teachers are faced with a difficult challenge: they must integrate the knowledge of pedagogical contents or the knowledge of the content that deals with the teaching process, the knowledge of the technological contents that refer to how technology can create new representations for the specific content, technological pedagogical knowledge which refers to the knowledge of how various technologies can be used in teaching. In the specific case, the integration of these elements refers to a Mathematics II course intended for students of the first year of Mechanical and Management Engineering.

### Instrumental Genesis

The idea of instrumental genesis proposed by Verillon and Rabardel (1995) seems an adapted framework to describe the use of Distance Learning in mathematics education. The instrumental genesis distinguishes an artifact (an artificial object/instrument) from an instrument (a psychological construct) by defining the instrument as a mixed entity composed of both components related to the characteristics of the artifact and subjective components (patterns of use) that come out from the Situated Instrumented Activity (Vérillon 2000) or the activity involving a subject (as a user), an artifact (as an instrument) and an object (as epistemic transformation, for example, the knowledge of functions in two variables). This hybrid entity takes the object into account and describes its functional use for the subject (Rabardel and Samurcay 2001) A scheme is a systematic procedure for using a given instrument to achieve a given purpose. Therefore, an instrument is a psychological construct in the field of cognitive ergonomics (Verillon and Rabardel 1995). The elaboration and evolution of instruments is a long and complex process that Rabardel calls instrumental genesis. It is articulated in two processes: instrumentalization, related to the appearance and evolution of the different components of the artifact, for example, the progressive recognition of its potentials and limits; instrumentation, related to the appearance and development of patterns of use. In our case, the didactic activities carried out with AR involved students (as users and protagonists of the didactic-educational path), technological objects such as 3D glasses, tablets, and PCs (as instruments) and an object, intended in an educational sense as a mathematical item to be recognized, internalized and contextualized (the real functions of two real variables). The teacher thus makes use of technological artifacts that undergo a triple process of instrumental genesis:

- from the didactic point of view, because their use is aimed at generating knowledge and enhancing skills;
- from the pedagogical point of view, because their use is subordinated to suitable teaching methods activated by the teacher and aimed at the construction of mathematical meanings;
- from a technological point of view, because the use of technologies is not an end but implements an effective mobilization of strategies aimed at learning.

### Fuzzy Cognitive Map

In this work, parameters related to interaction, participation and motivation, and their causal relationships, have been identified and analyzed through a Fuzzy Cognitive Map in order to describe the state of the student during the course. Kosko (1986) defines a Fuzzy Cognitive Map as a graph structure representing casual relationships. It can be used to describe complex systems/environments in a symbolic way, highlighting events, processes, and states. In an FCM, a node of the graph is called concept and an edge is called weight. The edge allows for implementing a causal relationship between two concepts, and the weight represents the strength of the influence of the relationship, described with a fuzzy linguistic term (e.g., low, high, very high, etc.). According to Codara (1998), FCMs can be used for various purposes, including:

- underline the behavior of agents, understand the reasons for their decisions and actions taken, highlighting any distortions and limits in their representation of the situation (explanatory function);
- predict future decisions and actions (forecasting function);
- help decision-makers to reflect on representation of a given situation (reflexive function).

A FCM can be formalized through a 4-tuple (*N*, *E*, *B*, *f*), where:

1. $N = {N_1, N_2,..., N_n}$ is the set of n concepts which are represented by the nodes of the graph.
2. $E: (N_i, N_j) \rightarrow e_{i,j}$ is a function $(NXN \rightarrow [-1,1])$ which associates the weight $e_{i,j}$ to the edge between the pair of concepts $(N_i, N_j)$;
3. $B: N_i \rightarrow B_i$ is the activation function which associates to each concept $N_i$ a sequence of activation values, one for each time instant $t: \forall t, B_i (t) \in [0,1]$ is the activation value of the concept $N_i$ at time *t*. $B(0) \in [0,1]^n$ is the initial activation vector containing the initial values of all the concepts; $C(t) \in [0,1]^n$ is the state vector at a certain time instant *t*.
4. $f: R \rightarrow [0,1]$ is a transformation function with a recursive relation for $t \ge 0$ between $B(t + 1)$ and *B*(*t*):
  1 $$\begin{aligned} \forall i \in \{1,...,n\}, B_i(t+1)=f(\displaystyle \sum \nolimits ^{n}_{\begin{array}{c} j=1\\ j\ne i \end{array}}e_{j,i} B_j(t)) \end{aligned}$$

As *f*(*x*), different types of functions can be used (the linear function, the sigmoid function or the bivalent function). FCM can be used to make a what-if inference, starting from a given initial activation vector *B*(0), to understand what will happen next to the modeled system/environment. Fuzzy Cognitive Map is developed by integrating existing experience and knowledge related to a system. This can be achieved by using a group of experts to describe the structure and behavior of the system under different conditions. With FCM it is usually easy to find which factor needs to be changed and being dynamic modeling tools, the resolution of the system representation can be increased by applying further mapping. The main reasons why FCMs are used are: easy to build and parameterize; easy to use; flexible in representation; easily understandable even to non-expert users; convenient for managing complex problems related to the processing and management of knowledge in a structured way; convenient for managing the feedback structure of the modeled system with dynamic effects.

## The Case Study

This section describes an embedded case study with both ideographic and nomothetic intentions (Yin 1981, 2013). Section 4.1 describes the context of the experimentation and the technological tools used to support distance learning. The methodology adopted is described in Sect. 4.2. Section 4.4.2 is devoted to the description of the FCM. Section 4.2.3 describes the sample under study and the reference population.

### Context

The didactic experimentation involved the students of the first year of Mechanical Engineering and Management Engineering at the University of Salerno, the course teacher, two experts of the discipline for the exercises, three students attending the master’s degree in engineering with the function of the tutor. The course was carried out during the second semester of the first year after students had attended and/or taken a Calculus 1 exam.

Because of the COVID-19 pandemic, after the first two face-to-face lessons, the teaching activities of Calculus II course were conducted through Distance-Learning and were articulated into Asynchronous Online Learning (AOL) and Synchronous Online Learning (SOL).

One of the teacher’s challenges was quickly adapting the didactic content plan with completely remote teaching. In order to motivate students, encourage participation and engagement, keep students’ degree of attention alive, stimulate their epistemic curiosity, interest, optimism, and passion, it was necessary to readjust the disciplinary methodological knowledge and teaching contents through the use of an adaptive e-learning platform. The course has been restructured in accordance with the constructive alignment suggested by Biggs and Tang (2010). The course included 90 hours of lessons (6 h per week), divided into 54 h of theory lessons and 36 h of training; also 24 h of exercises with the tutors, dividing the students into two sub-groups, 12 additional hours of exercises for students who have reported an insufficient evaluation at the first test. The course has been designed considering the indications of the Teaching Council and the Lisbon descriptors, as follows:

- Knowledge and understanding. The goals are understanding the terminology used in mathematical analysis; knowledge of demonstration methods; knowledge of the fundamental concepts of mathematical analysis. knowledge related to integral functions of a variable, numerical series, sequences and series of functions, functions of several variables, differential equations, multiple integration, curves and curves integrals, surfaces and surface integrals, vector fields.
- Appling knowledge, Understanding and Engineering Analysis. The goals are applying the theorems and the rules studied to solve problems; building methods and troubleshooting procedures; Know how to process and communicate information using a formal linguistic log; applying knowledge of the concepts and methods of calculus and mathematical tools to solve differential equations, integral curves, integral and surface integrals, perform series and integral calculations, calculate maximum and minimum functions of two variables; applying knowledge to develop demonstrations of certain theorems consistently.
- Applying knowledge, understanding and engineering design. The goals are applying knowledge to find the most appropriate methods to solve a math problem; be able to find optimizations in the process of solving a math problem.
- Making judgments and engineering practice. The goal is applying the acquired knowledge to contexts different from those presented during the course.
- Communication skills and transversal skills. The goal is learning more about the topics covered by teaching materials other than those proposed during the course.
- Learning skills and transversal skills. The goals are Learn how to decipher the topics discussed using teaching materials other than those proposed during the course; develop a positive attitude toward math based on respect for truth and availability to seek motivation and to clarify its validity.

The contents of the course have been scheduled as reported in Table 1.

**Table 1:** Contents of the course

Content	Hours of Theory	Hours of Exercise
Linear algebra	8	6
Differential equations	6	4
Functions of several variables	6	4
3D geometry, curves, and integral curves	6	4
Double integrals	6	4
Triple integrals	4	2
Differential forms	6	4
Surface and surface integrals	6	4
Function series	6	4
Total	54	36

A first difficulty in restructuring the course was to choose which essential elements, concerning both the didactic and methodological aspects and the mathematical contents, had to be transferred to the Distance-Learning approach. The contingency showed a need for a reorganization of the teaching-learning activities related to both thought and action, taking into account the student’s educational needs.

The following online resources have been used: custom adaptive e-learning platform (D’Aniello et al. 2020a), Microsoft Teams, Doceri, Edmodo, the teacher’s website, the teacher’s YouTube channel, Geogebra AR. Some of these have already been used in previous courses as a support for face-to-face teaching.

The use of these resources is in accordance with the framework proposed by Bray and Tangney (2016). This approach focuses on the creation of activities that fall within the Transformation space: transformative uses of technology are those which allow significant task redesign (modification) or permit the creation of tasks that would not be possible without the digital tools (redefinition). These aspects have contributed in the implementation of the activities of this course especially as regards the parameter of engagement in learning mathematics.

Furthermore, the lessons were delivered on the Microsoft Teams platform, keeping the time duration of the lessons and the teaching content unchanged, but providing for more breaks during the lesson and trying to interact as much as possible with the students. The exercise sessions were conducted by dividing the students into virtual subclasses so that they could interact with the colleagues in small groups, while the teacher could intervene in the various classes with coaching and scaffolding actions.

Often the students confronted each other about carrying out the exercises and pointed out the difficulties they encountered or commented on the other students’ performance. The teacher and tutors, in a non-invasive way, had the opportunity to read the comments and analyze the progress of the exercises. Very often, students, especially those who had more difficulties, sent private messages to the teacher or tutors. This in particular allowed us to monitor step by step the skills acquired by the students, the difficulties they encountered, the points to dwell on with more considerable attention.

Furthermore, on the teacher’s YouTube channel, video clips of the lessons and examples of exercises were uploaded. The Doceri app has been integrated into the Microsoft Teams platform and used as a digital board, also to create video clips with solved exercises.

The adaptive e-learning platform was used to provide students with the teaching material of the course both in a textual and interactive format, the assignments to be completed at the end of the topic, check the corrections of the exercises, etc. In addition, the integrated feedback system has allowed the teacher personalized teaching, centered on the needs of the individual student.

Some tools, among those described, were an integral part of the course already in previous years. The tasks assigned to the students described above have been designed with the objective to create the conditions for students to face problems from their own perspective and in an autonomous way. One of main goals was to create an online community of practice in which students were connected by a shared experience, in an attempt to give meaning to their learning experience.

### Methodology

The adopted methodology is based on a single case study to identify intervention strategies on the specific situation of didactic hardship that has arisen following the pandemic. Contrary to previous studies (De Witte and Rogge 2014; Ghavifekr and Rosdy 2015) in which the use of technologies was a choice of the teacher, in this case, however, the teacher was forced to use ICT as the only means of teaching. Direct observation, understood as student-teacher interaction through the use of information technologies, was a way to collect empirical data on the teaching effectiveness. The results obtained from the analysis conducted on the data available for completely remote teaching were compared with those obtained from the data collected in a previous experiment in which the course was conducted in blended mode, i.e., in the 2018–2019 academic year. The approach was both of an ideographic nature based on qualitative methods through a questionnaire to students and of a nomothetic nature based on quantitative data that emerge from the results of the tests, from the responses to the questionnaire based on the Likert scale (Likert 1932) and from students’ interactions with the e-learning platform. In fact, both in the academic year 2018/2019 and 2019/2020 the students who attended the Calculus II course were given a questionnaire to bring out the students’ beliefs on the difficulties encountered, on the most difficult topics, and the importance of the use of an e-learning system. The questionnaires included hierarchical questions in which the students ranked in order of importance a certain number of modalities of a phenomenon, based on the Likert scale and open questions through which the interviewee was free to express herself in the form she preferred; the former was designed so that the student’s emotional state could be inferred; the latter was formulated so that it was possible to deduce elements of appreciation and criticalities of the didactical approach adopted. The questionnaire was sent to the students via Google Forms. The multiple-choice ones have been set according to the Likert scale.For scaling responses in the survey, we used the format of a typical five-level Likert item, as shown in Table 2.

**Table 2:** Likert scales about statements of agreement, frequency, and satisfaction

Levels	(1)	(2)	(3)	(4)	(5)
Agreement	Strongly disagree	Disagree	Undecided	Agree	Strongly agree
Frequency	Never	Rarely	Sometimes	Often	Always
Satisfaction	Not at all satisfied	Slightly satisfied	Moderately satisfied	Very satisfied	Completely satisfied

Further qualitative information was collected from the analysis of the protocols derived from the dialogues between students, and between students and teachers on the e-learning system used. A specially created Fuzzy Cognitive Map summarizes both qualitative and quantitative data, in terms of participation, motivation, and engagement, comparing them with the data of the previous cohort. As for the analysis of the disciplinary skills, a comparison between the results of the first and second mid-tests of the 2018/2019 and 2019/2020 student cohorts was carried out, taking into account the evaluation grid, reported in Table 3.

**Table 3:** Grades and competence levels

Grade	Competence level
A	Advanced
B	High
C	Medium
D	Initial

#### Fuzzy Cognitive Map for Data Analysis

This section describes the data analysis technique, based on a Fuzzy Cognitive Map (FCM), we defined and implemented. The objective of the FCM is to consider all the effects that the variables identified in the Status Model, widely discussed by the authors in their previous works (Capone and Lepore 2020; D’Aniello et al. 2020a), have on the engagement, motivation and participation of the learner, which are the three high-level concepts representing the current status of the learner.

With respect to other fuzzy approaches that could be used to represent the situation model (like, for instance, Fuzzy Inference Systems with if-then rules) the use of FCM provides us with these advantages:

- FCMs are based on causal cognitive mapping, which provides an efficient way to elicit and capture knowledge of the experts of the domain and provide an intuitive way to represent such a knowledge which can be easily managed and updated by such experts Kokar and Endsley (2012);
- maps can be based on interviews, text analysis or group discussions and can be easily modified or extended by adding new concepts and/or relations or changing the weights assigned to causal links Kosko (1986), Kokar and Endsley (2012);
- FCMs have been extensively used as a way to support situation identification and decision making, helping decision-makers in gaining a better understanding of the domain, of the situation and improving their mental models Jung and Lee (2018);
- traditional FIS could require a high number of rules to represent complex relations, especially when a high number of inputs needs to be considered Abeer and Miri (2014).

**Fig. 2:**
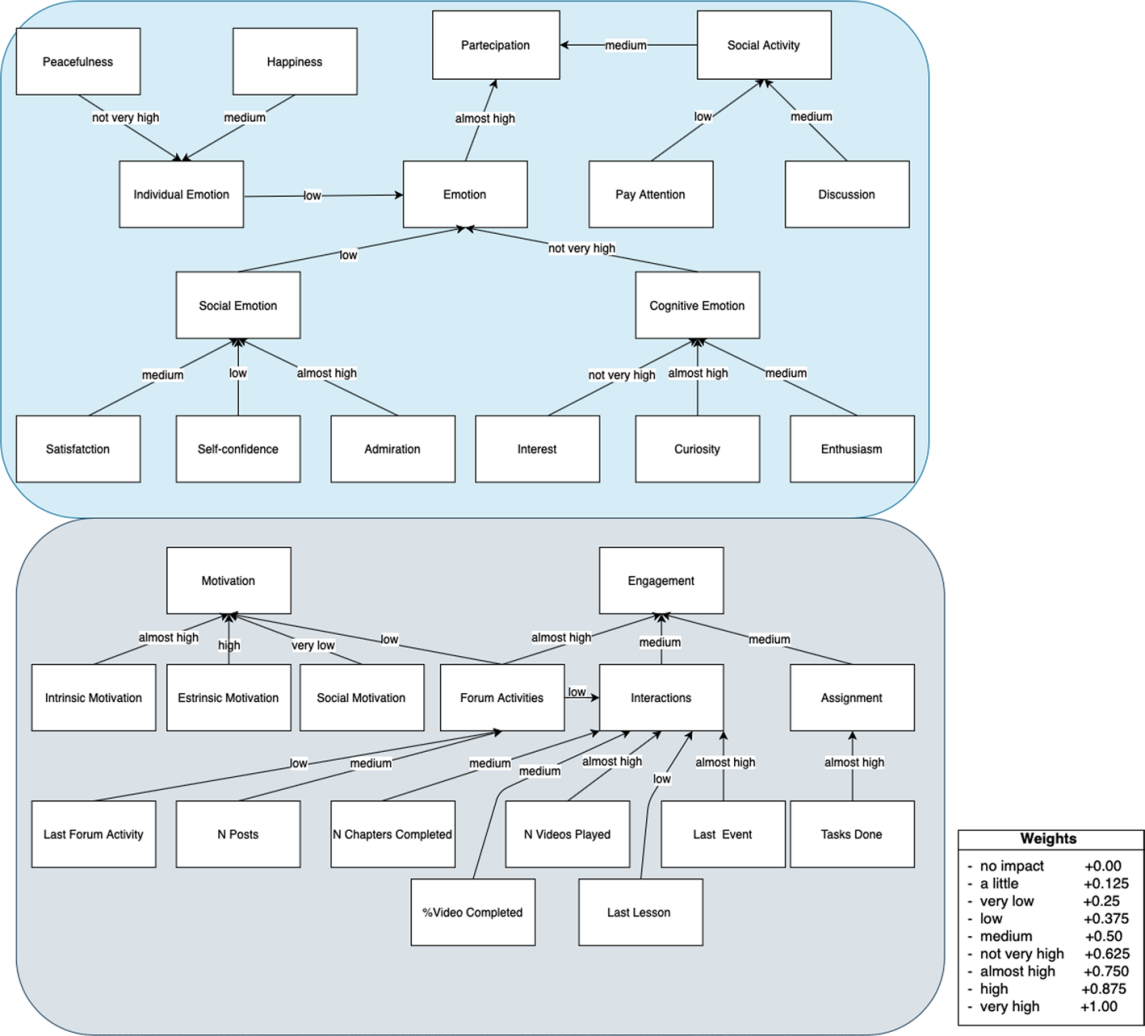
Fuzzy cognitive map as status model

The FCM has been defined by a team of four experts. Each expert, starting from the status model we have defined has proposed his FCM to identify the causal relationships and the weights existing between the available concepts. The weights are represented by seven linguistic terms: no impact = 0.00, very low = 0.165, low = 0.335, medium = 0.50, almost high = 0.665, high = 0.835, very high = 1.00. Then, we aggregate the different maps proposed by the experts to obtain one FCM. When some differences arise between the relationships and weights proposed by the experts, we asked them to discuss these differences and try to find an agreement, until they achieve a sufficient degree of consensus. This allowed us to obtain the FCM in Fig. 2.

The FCM can be considered as organized in different layers. The low layer contains the concepts of the FCM representing the variables discussed in (Capone and Lepore 2020; D’Aniello et al. 2020a), and which are partially listed in the Fig. 2 for reasons of legibility. The activation levels of these “leaves” concepts represent the value of each variable. When a value of these variables changes (due to the actions performed by the learner), the other concepts of the FCM are influenced according to the causal relationships between them. The middle layer contains the concepts composing the engagement-motivation (Interaction, Assignment and Forum Activities) and participation (Emotion, Social Activity).

The final layer contains the concepts Engagement, Motivation and Participation representing the current learner status. These concepts are influenced by the concepts of the middle layer.

The activation values of the concepts of the middle and final layers are computed, starting by the activation levels of the input layer, using the inference process of the FCM. Specifically, the activation level of the concept can be iteratively calculated:

2 $$\begin{aligned} B_i(t+1)=f(B_i(t) + \displaystyle \sum \nolimits ^{n}_{\begin{array}{c} j=1\\ j\ne i \end{array}}e_{j,i} B_j(t)) \end{aligned}$$

where $B_i(t+1)$ is the activation value of concept $B_i$ at time $t+1$ , $B_j(t)$ is the activation level of the concept $B_j$ at time *t* , $e_{j,i}$ is the weight between concept $B_j$ and $B_i$, *f*() is a transformation function. In this work, we used a linear function for *f*():

3 $$\begin{aligned} B_i(t+1)=\alpha (B_i(t) + \displaystyle \sum \nolimits ^{n}_{\begin{array}{c} j=1\\ j\ne i \end{array}}e_{j,i} B_j(t))\end{aligned}$$

where $\alpha$ is a real number.

#### Definition of the Sample

To perform the experiment, the data of interest were collected and analyzed in the classes of Calculus II of Mechanical/Management Engineering at the University of Salerno in the academic years 2018/2019 and 2019/2020. The 2018/2019 course was held in person through blended teaching, alternating traditional teaching with the use of technological tools such as an adaptive e-learning platform. The course of the year 2019/2020, on the other hand, took place according to the methods illustrated in Sect. 4.1.

The Engagement parameters have been made available by the systems used through the collection of student interactions. The parameters relating to Motivation were determined through the analysis of questionnaires administered during the course. The parameters related to Participation or Emotions and social activities are established through sentimental analysis on the video streaming of the webcams filming the students and through the answers to the questions in the questionnaire.

The data provided by the system for the different parameters of interest are analyzed through FCM execution to create the student’s status; by comparing the parameters of the participants it will be possible to understand if the use of completely remote teaching did not negatively affect the status of students.

The two classes are made up of 131 and 112 students respectively. For the experimentation, 60 samples from the first group and 60 samples from the second group were randomly selected. Cochran’s formula was used to calculate the sample size:

4 $$\begin{aligned} n_0=\frac{Z^2pq}{e^2} \end{aligned}$$

Where

- *e* is the desired level of precision (i.e. the margin of error);
- *p* is the (estimated) proportion of the population which has the attribute in question;
- *q* is $$1 - p$$;
- the z-value is found in a Z table. It is the abscissa of the normal curve that cuts off an area $\alpha$ at the tails ($1 - \alpha$ equals the desired confidence level, e.g., 95%);
- $n_0$ is the sample size.

In our experimentation the chosen parameters were: ${Z = 99\%; p = 0.90; e = 0.10}$.

## Results and Discussion

In this section, data from a qualitative analysis (Sect. 5.1) and a quantitative analysis (Sect. 5.2) are reported highlighting main research findings.

### Qualitative Analysis

From the questionnaire sent to the students (reported in Appendix A) and the social dialogues between students and between students and teachers on the e-learning system, information was obtained regarding the parameters of participation, engagement, and motivation.

The students were asked to comment on the Calculus II course they attended, highlighting how involved they were in the teaching activities, how motivated they were taking the course, studying, and their feelings about the course.

In the next subsections are reported some answers from students who followed the course in a blended mode in the 2018/2019 academic year and in remote mode in the 2019/2020 academic year, to highlight the aspects of the three parameters under analysis. Specifically, in the Table 4 are reported the students’ answers grouped by the covered parameter.

**Table 4:** Students’ answers grouped by the covered parameter

Student parameter	Students
Motivation	S1, S2, S6, S7, S13
Engagement	S3, S12
Participation	S4, S5, S8, S9, S10, S11

#### Answers Related to Motivation

In this subsection are reported the students’ answers in which it is possible to extract information on their motivation.

*S1: I particularly appreciated the teacher’s approach to the subject and the time dedicated to classroom exercises to clarify any doubts. The time set for the lessons certainly did not help in terms of attention, but in most cases, however, the lesson went on without problems.*

*S2: It cannot be denied that the Calculus II course is complex, but I can say that the lessons were almost always clear especially from the point of view of the exercises discussed in class with the teachers. Overall, it was a course that I enjoyed following considering the difficulties inherent in the subject studied.*

As can be seen from the words of students S1 and S2 (2018/2019), the dialogue in presence with the teacher and the exercises were a good motivation to follow the course. This opinion is common to many other students.

Here are some of students’ answers who followed the course in remote mode in the academic year 2019/2020.

*S6: Now I am distressed by everything around me; there is the fear of not being able to return to our lives and all this still does not seem true to me. I am not comfortable following the online lessons on the e-learning platform.*

*S7: I would never have taken a fully online course. I prefer contact with colleagues and teachers even if we have been enabled to study in the best possible way with the considerable volume of teaching material and exercises available on the platform.*

*S13: In this period of social isolation, studying has become a reason for me not to think about the current state but to have future perspectives.*

Students S6 and S7 highlighted the difficulties deriving from a critical emergency, revealing that the only reason they agreed to take an online course is that they were forced to. This concept is expressed by almost all students. These attitudes seem to show a greater extrinsic motivation than the intrinsic and social one, as confirmed by the analysis of quantitative data. Furthermore, from some answers received, such as that from student S13, students seem to be motivated to improve their skills not only for the purposes of grades but to have better future prospects.

#### Answers Related to Engagement

In this subsection are reported the students’ answers in which it is possible to extract information on their engagement.

*S3: The course was well organized with extra material usable by e-learning helpful for carrying out the tests, with exhaustive and complete exercises carried out in the classroom. Enough exercises have been available online to keep practicing.*

The S3 student (2018/2019) highlights how the e-learning platform is essential to deepen the theoretical aspects through extra didactic material and to be able to constantly exercise in assigned tasks.

*S12: My feelings are positive, the course worked well because in addition to the didactic material always available on the e-learning platform, the teacher was able to integrate the lessons with an interactive whiteboard which is fundamental for this subject. Great to have also the recordings of the lessons available.*

The words of student S12 (2019/2020), also shared by other students, reveal how the use of the platform allows using in a structured way the considerable digital content to support teaching both in terms of theoretical and practical aspects.

#### Answers Related to Participation

In this subsection are reported the students’ answers in which it is possible to extract information on their participation.

*S4: The course was made very interesting thanks to the many examples of mathematical applications to physics.*

*S5: I got hooked on the subject thanks to the high level of challenge presented by the exercises. Thanks to the support of colleagues and teachers it was sustainable.*

S4 and S5 students (2019/2020) underline how the content of the lessons and social interactions have played a fundamental role in keeping high the students’ interest and in keeping positive the feelings towards the subject.

*S8: My concerns have concerned both not being able to compare with colleagues in class, perhaps even at the end of the lesson, as I have always done before and certainly also doing the same with the teacher.*

*S9: The main concerns were the impossibility of having a direct confrontation with the teachers, poor communication between students and teachers, a greater detachment between students.*

*S10: I solved the organizational aspect, which is very important to me. However, I think I should still work on concentration, as in this mode and being in a home environment it tends to be inferior.*

*S11: A piece of advice I would give is to divide the lessons with breaks maybe every 45 minutes, to make them less heavy since it is frustrating to be at the PC all day.*

From what was reported by students S10 and S11, using completely remote teaching negatively affects their emotional state leading to drops in concentration and interaction. Furthermore, student S8 expresses the concern of not being able to compare himself with his peers. The same thing highlights student S9: the lack of direct confrontation seems to negatively affect the emotional state of the students. Those students (s8, S9, S10, S11) followed the course in remote mode in the academic year 2019/2020.

### Quantitative Analysis

In this subsection, the quantitative results of the experimentation conducted are shown. A first global observation showed that physical distance led students to participate more actively in educational proposals. For example, we have noticed that the interaction between students, through the social platform, has been more frequent than in previous years. In fact, the average number of accesses to the platform per single student was much higher this year: 12 in 2018/2019 and 57 in 2019/2020. The average number of students who attended the lectures was approximately the same, in 2018/2019 they were 45 out of 60, while in 2019/2020 they were 42 out of 60.

In addition, from the hierarchical questions it emerges that in the year 2019–2020, 69.77% of students answered 4 or 5 on the Likert scale to the question of how frequently they confronted each other on the teaching activities of the course; while in 2018–2019 40%. 92% of the students declared that they had interacted on the e-learning platform forum either assiduously or very frequently (4 or 5 on the Likert scale) in the year 2019–2020. In fact, as they themselves declared, the forum allowed them to recreate the environment of the study room, although virtual, in which to discuss the solution of the exercises proposed in class. In 2018–2019, however, only 25%.

As a further element of qualitative analysis, it was decided to compare the data that emerged from the tests during the two academic years, shown in Table 5.

**Table 5:** Results of the tests done by students during the two academic years

Mark	First mid-term test 2018/2019	First mid-term test 2019/2020	Secondo mid-term test 2018/2019	Second mid-term test 2019/2020
A	4%	6%	15%	10%
B	12%	17%	18%	15%
C	31%	30%	23%	31%
D	22%	19%	12%	15%
Fail	32%	25%	32%	28%

A less critical situation seems to emerge from the data in Table 5 than that which emerged from the qualitative data. In fact, there is no statistically significant difference between the two data; indeed, it is possible to note that the percentage of those who failed the tests in 2019/2020 is slightly lower than that of 2018/2019. A possible interpretation of these data is suggested by having completely restructured the didactic activities. In fact, Technological Content Knowledge, Pedagogical Content Knowledge, and Technological Pedagogical Knowledge have been intersected in order to convey the specific contents of the discipline through the use of alternative teaching methodologies supported by the use of technologies. The use of technologies has undergone an elaboration and evolution of students through a process of instrumental genesis: students have progressively realized the potential of e-learning but also its limits.

From the qualitative and quantitative data that emerged, the input values were extrapolated for the Fuzzy Cognitive Map (FCM), used to derive the students’ levels of the engagement, motivation, and participation parameters. To verify if the obtained results are statistically significant, we performed an ANOVA test, comparing two groups: Blended Learning (students who attended classroom lessons and used the supporting e-learning platform) and Distance Learning (students who followed the course completely online). We obtained the following results for the F-test statistics, considering a significance level alpha = 0.05:

- Considering the level of participation, F-critic = 7.79 and *p*-value = 7.04;
- Considering the level of engagement, F-critic = 9.01 and *p*-value = 8.83;
- Considering the level of motivation, F-critic = 8.59 and *p*-value = 7.91.

**Fig. 3:**
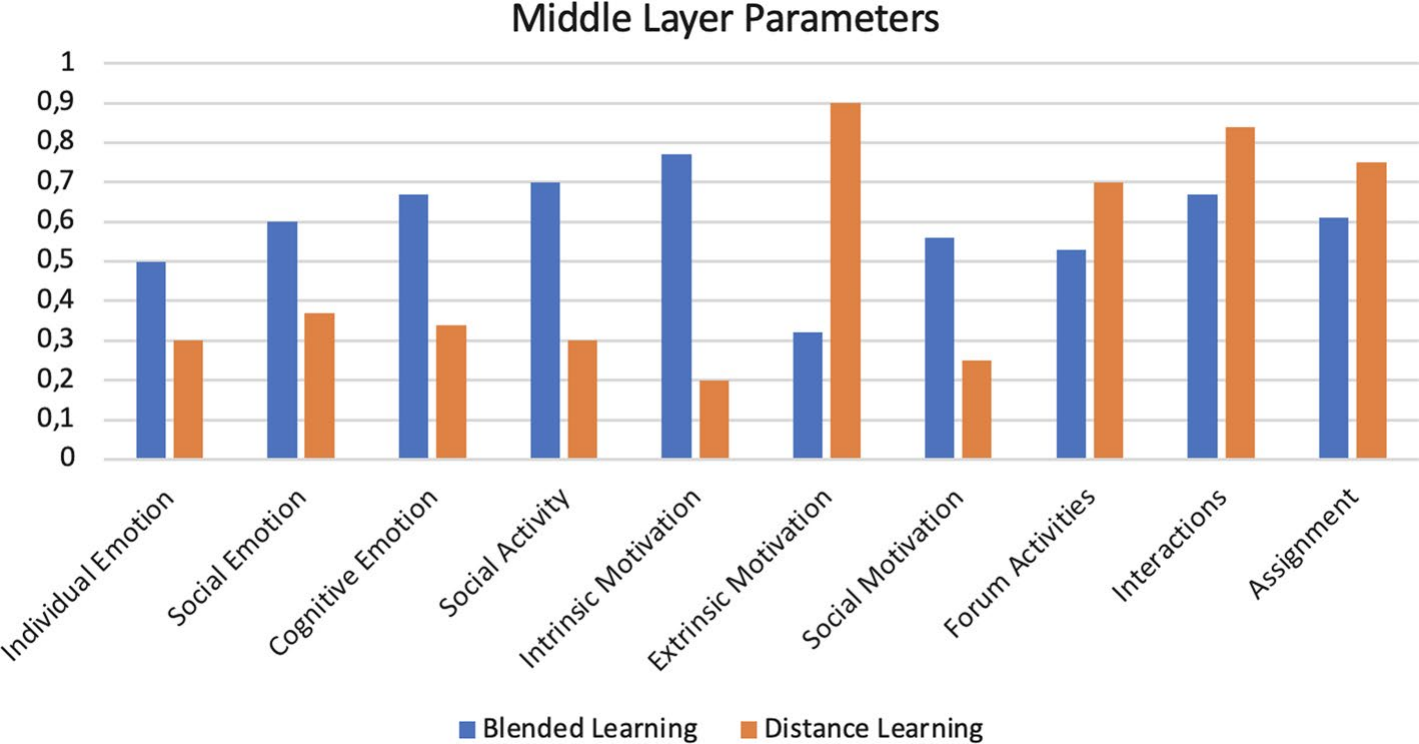
Middle layer results

**Fig. 4:**
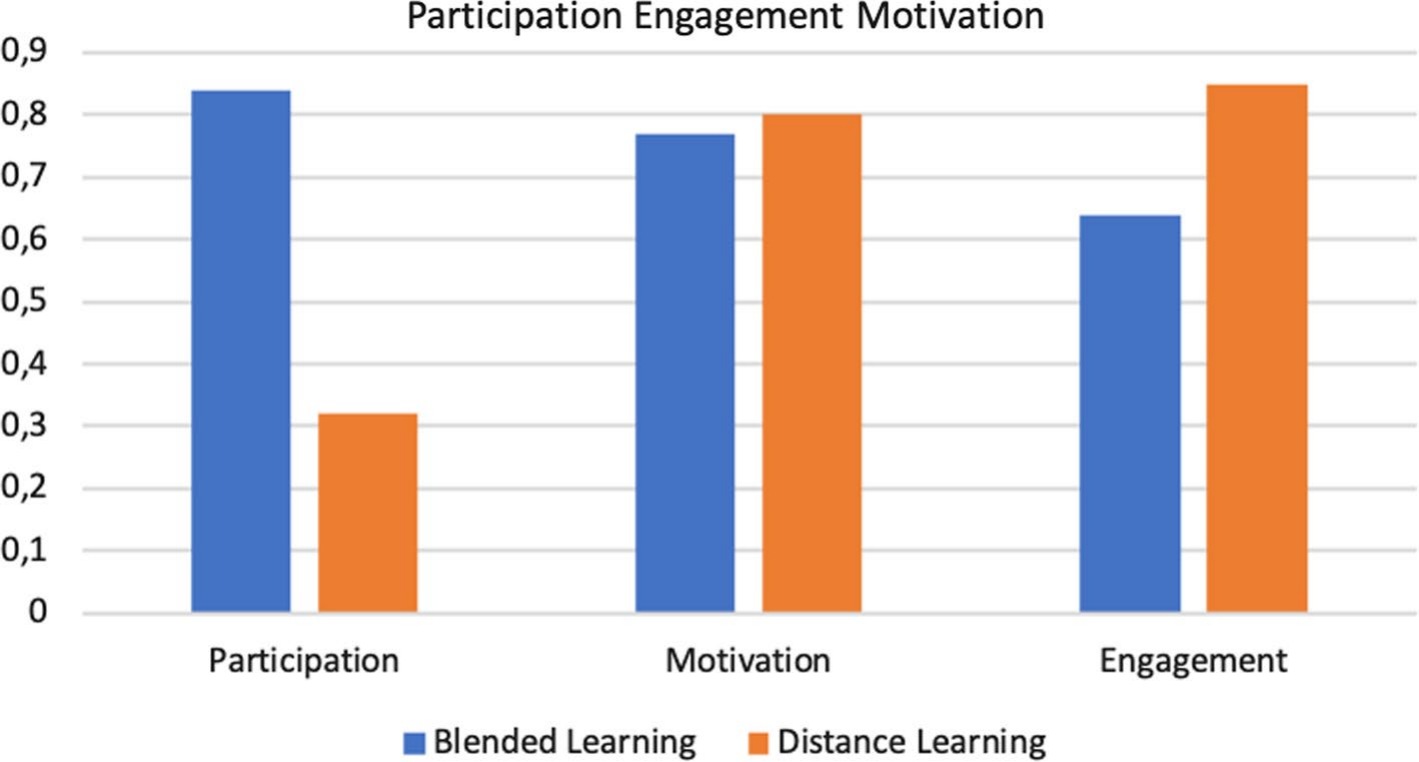
Participation, engagement and motivation results

Consequently, the tests demonstrate that the results are statistically significant. The results obtained are schematized through Figs. 3 and 4.

Figure 3 shows the average input values for the middle layer FCM concepts, shown in Fig. 2 and described in Sect. 4.2.2. The two graphs show the comparison between the two groups of students into which the sample was divided: “Blended Learning” (represented in blue), students who attended classroom lessons and used the reference e-learning platform, while in “Distance Learning” (shown in orange) there are students who have followed the course completely online.

The first three parameters reported, namely Individual Emotion, Social Emotion, and Cognitive Emotion, which refer to the emotional states of students (Peacefulness, Happiness, Satisfaction, Self-confidence, Admiration, Interest, Curiosity, Enthusiasm, Pay Attention, Discussion) highlight a situation of greater positivity in the 2018/2019 academic year in which teaching was of the blended type. The emotional state of the students of the academic year 2019/2020 was affected by the emergency caused by the pandemic. This affected both group and cognitive emotions and is consistent with the state of frustration, fear, sometimes alienation manifested by the students through the questionnaire.

The Social Activity parameter, the result of the inference on Pay Attention and Discussion levels, presents a significant difference between the two academic years. This could be motivated by the difficulty of the students to pay attention during the online lesson as shown by some answers to the questionnaire. The teachers themselves found themselves for the first time in the situation of providing completely remote teaching and most likely they were not always able to regulate lesson times and encourage social dialogue.

The parameters related to motivation (Intrinsic Motivation, Extrinsic Motivation, and Social Motivation) show a notable difference between the two years. Specifically, in the 2018/2019 academic year, students took part in blended activities because they were driven by the desire to enrich themselves and the desire to share this experience with colleagues. While the students of the academic year 2019/2020 had an external constraint to follow the online course as the only way to obtain the attendance required to access the final exam.

Forum Activities, Interactions and Assignment, or the parameters related to Engagement, show balanced levels over the two years. Having the e-learning platform as the only tool available to access the teaching material and to carry out the exercises, the students of the academic year 2019/2020 show, even if slightly, a higher level of interaction than the students of 2018/2019 where the use of the platform was an additional part, used to deepen or to carry out some extra exercise compared to what was done in the presence.

**Fig. 5:**
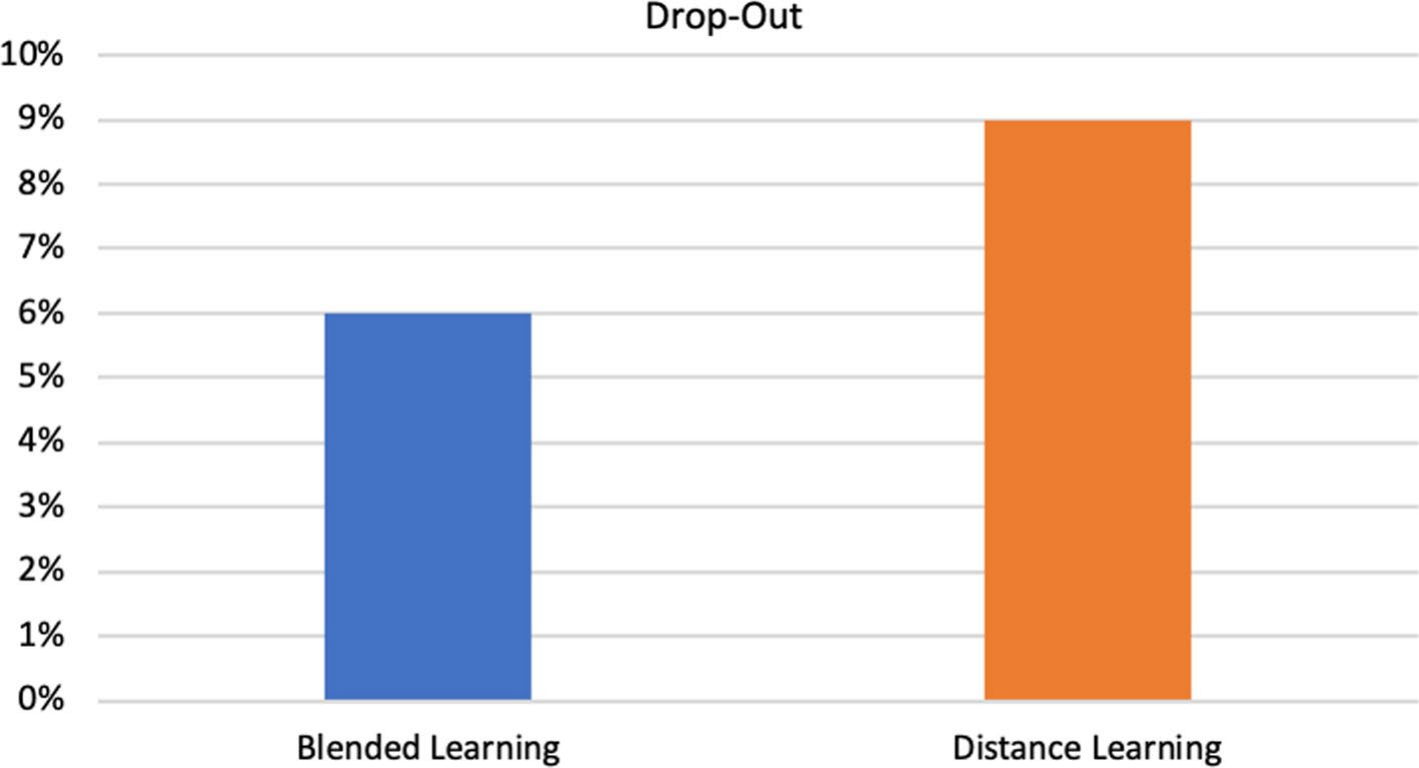
Dropout results

The average levels of engagement, motivation, and participation calculated through the execution of the FCM are shown in Fig. 4, which summarize what is reported in the analysis of the data of the middle layer parameters. The decline in student participation in the year 2019/2020 compared to the previous year seems to be due to the negative impact of the emergency for the pandemic on the emotional state of students. In addition, the sense of alienation and frustration has led to a loss of concentration and poor social activity. The level of motivation is comparable, but as noted, for students of the year 2018/2019 it is determined by intrinsic and social motivation, for students of the year 2019/2020 by an external tax. The engagement is, albeit slightly, greater for students of the academic year 2019/2020 who have massively used the e-learning platform as the only tool to follow the course and access the content made available by the teachers. Finally, the drop-out graph shown in Fig. 5, shows how the two values are comparable (6% in the year 2028–2019 and 9% in the year 2019–2020) and how they remained below an acceptable threshold. The use of an adaptive e-learning platform and the restructuring of educational content through the use of technologies, which led students to have a high level of engagement for the duration of the course, seem to have contributed to containing the phenomenon of drop-out feared at the beginning of the course through distance learning methods.

## Discussion

In this section, we will discuss the main research findings taking into account the research questions reported in the introduction of the paper. In addition, we will show the results gathered from our work highlighting the practical, methodological and theoretical implications.

The qualitative analysis based on the students’ answers of the year 2018/2019 shows considerable enthusiasm for using an e-learning platform as a tool for continuous comparison with teachers and colleagues and as a support to study. The same enthusiasm is not shown by the students of the year 2019/2020 who found themselves using e-learning as the only learning methodology while noting its usefulness as the only means of interaction with teachers. Students show discomfort both for the emergency situation and for the more technical aspects of the course setting. As can be seen from the qualitative data, participation, engagement, and motivation are altered by the emergency situation and the distance learning used.

These qualitative data are also confirmed by the quantitative analysis conducted through the analysis of questionnaires using the Likert scale and intersected by the summary ones obtained from the execution of the FCM.

From these two types of analysis, we gathered one of the most important practical implications of our research: on one hand Distance Learning is excellent as an additional and support methodology but, on the other hand, it highlights the ineffectiveness of completely remote teaching. Therefore, we propose, as a methodological implication of our research, a teaching method that integrates moments of distance teaching (carried out on digital platforms) with activities carried out in the presence, in the classroom, or in other university environments. A mix of styles, a fluid flow of knowledge between the physical classroom and the virtual classroom. We will call this Integrated Digital Learning.

Furthermore, from the quantitative analysis conducted on the data from the questionnaires administered to students, two important aspects can be noted:

- the students tried to make up for the physical presence by trying to interact constantly on the e-learning platform forum.
- the state of mind of the students influenced the study.

From qualitative and quantitative data analysis alike, it seems that behavioral engagement is also positively associated with the realistic, practical and guided discovery aspects of the task design, the activity structure and the transformative use of mobile technology; additionally, the impact of working in a team also appears to have had a positive effect. Mathematics confidence is positively associated with real, guided and practical tasks, with use of technology also appearing influential. Not surprisingly, the use of technology, both transformative and computational, is most significantly related to confidence using technology, with the variety of technologies noted as adding to flexibility and adaptability. The transformative and computational use of technology, in conjunction with the task design, appears to have the most influence on students’ attitude to using technology for learning mathematics.

Another important practical implication obtained from this analysis is that motivation, participation and engagement are altered by the emergency situation and the distance learning used. The motivation went from intrinsic to extrinsic given the external constraint to take the online course as the only way to obtain attendance. Participation plummeted because the emotional state and the desire for socializing were adversely affected. Engagement increased as a result of massive use of adaptive learning platform and of all the technological tools as the only means to follow the course and feel part of a community; however, by integrating Technological Content Knowledge, Pedagogical Content Knowledge, and Technological Pedagogical Knowledge to the use of an adaptive e-learning platform, students have reached adequate levels of competence.

To summarize, in response to the first research question, i.e. *“How do engagement, motivation, participation change with completely remote teaching?”*, it can be stated that for the year 2019/2020 the motivation of students became mainly extrinsic because following the course completely online was the only possible choice; this was also perceived as a heavy constriction. Participation has heavily undergone this change, in fact the students have experienced a period of alienation in which their feelings and their desire for sociality have been negatively influenced. On the other hand, after the first moments of bewilderment and uncertainty, the students tried to react and cope with this emergency situation, thanks to how the teachers have completely rethought their way of teaching by putting together Technological Content Knowledge, Pedagogical Content Knowledge and Technological Pedagogical Knowledge. This has resulted in an increase in student engagement as they have massively used the digital tools available as the only means to carry on their studies and feel part of a community.

In addition, in response to the first research question, namely *“Can an adaptive e-learning system and personalized teaching contribute to effective teaching, even if completely remote, in terms of competences acquired by students?”*, it can be stated that the role of the teacher in integrating the use of an e-learning platform in personalized teaching was fundamental. Therefore, a theoretical implication that emerges from this work is that the networking of the two theoretical frameworks, namely the TPCK framework and the instrumental genesis, has led the technological artifacts to undergo a triple process of instrumental genesis:

- from the didactic point of view, because adaptive e-learning system favored an improvement in skills;
- from the pedagogical point of view, because their use favored the construction of mathematical meanings;
- from a technological point of view, because the use of technologies implemented an effective learning strategies mobilization.

## Conclusions

Distance learning, already widespread in recent years, has taken on a new meaning in this particular pandemic moment. It has taken on the role of didactics of proximity, or rather didactics of being there, understood as openness to the world, as a willingness to enter into a relationship with things and with others, as a disposition that man freely assumes with respect to reality. It was indispensable for strengthening the web of relationships between teachers and students, and between teachers. This teaching of proximity has required the call to the functionalization of the didactic contents not as an end in themselves but aimed at an effective learning process, at the recovery of the educational value, and not merely in the accounting of the evaluation. Distance Learning could lead to unexpected connections that are not just accessing a server from a client through protocols or software but could rather favor synaptic connections that produce cognition and generate positive emotions that do not make us become victims of an emotional abduction and transported by fear, by anguish in a non-adaptive way.

This research work focused on distance learning and the virtual environment during the Covid-19 pandemic, highlighting how motivation, participation, and engagement are affected. The experimentation was conducted in a Calculus II class of the Engineering degree course with both an ideographic and nomothetic approach. The purpose of the experiment was to verify how the use of completely remote teaching influences the student’s status in terms of participation, interaction, engagement. A student with a high level of these indices proved to be more motivated in the study of the discipline positively influencing the improvement of skills. It was possible to understand that the use of completely remote teaching did not negatively influence the student’s status, assessed through FCM, and allowed satisfactory results to be achieved also in terms of acquired skills. The drop-out was contained within acceptable values.

The strategies implemented by the lecturers of the course aimed at integrating Technological Knowledge, Pedagogical Knowledge, and Content Knowledge, in accordance with the Technological Pedagogical Content Knowledge (TPCK) framework. Finally, the processes of instrumentation and instrumentalization have caused an instrumental genesis of technological artifacts.

Data were collected with a multi-method. In fact, qualitative data was collected from the analysis of an open-ended questionnaire. Through this questionnaire, students were able to freely express their ideas on the emergency they experienced, and this was important to have an overview of how the students were experiencing the situation in emotional terms. Quantitative data were collected through hierarchical questions based on a Likert scale with more targeted questions aimed at analyzing more specific areas such as engagement, participation, and motivation. Other quantitative data concerned the level of competence achieved by students in the tests and the number of accesses made on the platform. The qualitative and quantitative data were summarized through the three parameters of interest (engagement, participation, and motivation) calculated through an ad hoc FCM map.

The main findings are:

- Distance Learning is excellent as an additional and support methodology but highlight the ineffectiveness of completely remote teaching;
- motivation, participation and engagement are altered by the emergency situation and the distance learning used. The motivation went from intrinsic to extrinsic given the external constraint to take the online course as the only way to obtain attendance. Participation plummeted because the emotional state and the desire for socializing were adversely affected. Engagement increased as a result of massive use of adaptive learning platform and of all the technological tools as the only means to follow the course and feel part of a community;
- by integrating Technological Content Knowledge, Pedagogical Content Knowledge, and Technological Pedagogical Knowledge to the use of an adaptive e-learning platform, students have reached adequate levels of competence.

It is not certain whether these same results can be confirmed in a prolonged period of distance learning. The hope of the authors is to undoubtedly return to face-to-face teaching, where human values and relationships take on a concrete and visible form while trying to exploit the potential of distance learning, integrating the strengths of the two methodologies. At the moment this is not a possibility, given that the emergency situation is continuing without knowing how much longer this will proceed but the findings of this case study provide a fresh perspective and sets the stage for future, related research. In fact, the authors are working on modifying the adaptive e-learning platform used, integrating appropriate teaching methodologies to maintain high levels of engagement participation and motivation even in the long term. To support this progress, agile methodologies typical of software engineering are being tested both for platform development and for teaching. Finally, distance learning offered opportunity to rethink educational action from the point of view of contents, methodologies and student-teacher interactions alike. At the end of the pandemic, as next step, we think that some positive aspects of distance learning can be integrated with traditional teaching and enhance a blended modality of didactic action that can be called Integrated Digital Learning.

## Data Availability

The data presented in this study are available on request from the corresponding author. The data are not publicly available due to privacy reasons.

## Appendix A

See Table 6.

**Table 6:** Questionnaire provided to students useful for quantitative and qualitative assessment

#	Question	Answer				
1	Name and surname					
2	Gender	[ ] Male [ ] Female [ ] Other				
3	Enrollment	[ ] First year [ ] Previous years				
4	How frequently did you attend classes in the first semester?	Never [ ] 1 [ ] 2 [ ] 3 [ ] 4 [ ] 5 Always				
5	How frequently are you taking the mathematics II course?	Never [ ] 1 [ ] 2 [ ] 3 [ ] 4 [ ] 5 Always				
6	How much do you prefer to follow the lessons in synchronous mode?	Definitely Not [ ] 1 [ ] 2 [ ] 3 [ ] 4 [ ] 5 Definitely				
7	How much do you prefer to follow the lessons in asynchronous mode?	Definitely Not [ ] 1 [ ] 2 [ ] 3 [ ] 4 [ ] 5 Definitely				
8	How frequently do you use the e-learning platform?	Never [ ] 1 [ ] 2 [ ] 3 [ ] 4 [ ] 5 Always				
9	How frequently do you participate in the educational activities proposed on the social platform?	Never [ ] 1 [ ] 2 [ ] 3 [ ] 4 [ ] 5 Always				
10	How often do you compare yourself with your classmates on the didactic activities of the course?	Never [ ] 1 [ ] 2 [ ] 3 [ ] 4 [ ] 5 Always				
11	In your opinion, what is the overall quality of the course you are following	Very poor [ ] 1 [ ] 2 [ ] 3 [ ] 4 [ ] 5 Excellent				
12	What motivated you to attend the Mathematics II course?					
13	After reflecting on the questions above, express in a few lines your opinion on the teaching of Mathematics II, highlighting your feelings, positive and negative aspects					
14	How would you improve the course? Please provide any suggestions you have for improving both the teaching aspect and organizational aspects					
15	How did you react as soon as you learned that you could no longer attend classes? What aspects frightened you right away, what were your biggest concerns at the beginning?*					
16	What aspects of traditional teaching do you consider essential to your education and which you do not find in online teaching? *					
17	Which aspects of online education do you think can be maintained after the end of the Covid19 emergency? *					

The questions are almost identical for the academic years 2018/2019 and 2019/2020, except for some extra questions reported with asterix for the academic year 2019/2020

## References

[CR1] Abeer W, Miri B (2014). Students preferences and views about learning in a MOOC. Procedia-Social and Behavioral Sciences.

[CR2] Anthony, B., Kamaludin, A., Romli, A., et al. (2020). Blended learning adoption and implementation in higher education. In: *A Theoretical and Systematic Review. Tech Know Learning*

[CR3] Baudrillard, J. (1992). Pataphysics of year 2000. Originally published in French as part of Jean Baudrillard, L’Illusion. de la fin: ou la greve des evenements, Galilee: Paris, 1992 (Charles Dudas, Trans.) York University, Canada

[CR4] Bergmark Ulrika, Westman Susanne (2018). Student participation within teacher education: emphasising democratic values, engagement and learning for a future profession. Higher Education Research and Development.

[CR5] Biggs, J., & Tang, C. (2010). Applying constructive alignment to outcomes-based teaching and learning. In: *Training material for “quality teaching for learning in higher education” workshop for master trainers, Ministry of Higher Education, Kuala Lumpur* (pp. 23–25)

[CR6] Biggs JB (2011). Teaching for quality learning at university: What the student does.

[CR7] Branchetti L, Capone R, Tortoriello FS (2018). Unesperienza didattica half-flipped in un ambiente di apprendimento SCALE-UP. Annali online della Didattica e della Formazione Docente.

[CR8] Bray A, Tangney B (2016). Enhancing student engagement through the affordances of mobile technology: a 21st century learning perspective on Realistic Mathematics Education. Mathematics Education Research Journal.

[CR9] Capone, R., & Lepore, M. (2020). Augmented reality to increase interaction and participation: A case study of undergraduate students in mathematics class. In: *International Conference on Augmented Reality, Virtual Reality and Computer Graphics* (pp. 185–204). Springer, Cham

[CR10] Capone, R., De Caterina, P., & Mazza, G. (2017). Blended learning, flipped classroom and virtual environment: challenges and opportunities for the 21st century students. In: *Proceedings of EDULEARN17 Conference* (pp. 10478–10482)

[CR11] Capone, R., Del Regno, F., & Tortoriello, F. (2018). E-Teaching in mathematics education: The teacher’s role in online discussion. *Journal of e-Learning and Knowledge Society,**14*(3)

[CR12] Capone R, Adesso MG, Del Regno F, Lombardi L, Tortoriello FS (2021). Mathematical competencies: a case study on semiotic systems and argumentation in an Italian High School. International Journal of Mathematical Education in Science and Technology.

[CR13] Chang M, DAniello G, Gaeta M, Orciuoli F, Sampson D, Simonelli C (2020). Building ontology-driven tutoring models for intelligent tutoring systems using data mining. IEEE Access.

[CR14] Codara, L. (1998). * Le mappe cognitive: uno strumento di analisi per la ricerca sociale e per l’intervento organizzativo*. Carocci

[CR15] D’Aniello, G., De Falco, M., Gaeta, M., & Lepore, M. (2020a). A situation-aware learning system based on fuzzy cognitive maps to increase learner motivation and engagement. In: *Proceedings of the 2020 IEEE International Conference on Fuzzy Systems (FUZZ-IEEE)* (pp. 1–8)

[CR16] D’Aniello, G., de Falco, M., Gaeta, M., & Lepore, M. (2020b). Feedback generation using Fuzzy Cognitive Maps to reduce dropout in situation-aware e-Learning systems. In: *Proceedings of the 2020 IEEE Conference on Cognitive and Computational Aspects of Situation Management (CogSIMA) (pp. 195–199). IEEE*

[CR17] De Freitas SI, Morgan J, Gibson D (2015). Will MOOCs transform learning and teaching in higher education? Engagement and course retention in online learning provision. British Journal of Educational Technology.

[CR18] De Witte K, Rogge N (2014). Does ICT matter for effectiveness and efficiency in mathematics education?. Computers and Education.

[CR19] Dominguez, R. G. (2012). Participatory learning. *Encyclopedia of the Sciences of Learning,* 2556–2560

[CR20] Faggiano E, Ferrara F, Montone A (2017). Innovation and Technology Enhancing Mathematics Education.

[CR21] Finn JD, Zimmer KS (2012). Student engagement: What is it? Why does it matter?. Handbook of research on student engagement.

[CR22] Fredricks JA, Blumenfeld PC, Paris AH (2004). School engagement: Potential of the concept, state of the evidence. Review of Educational Research.

[CR23] Gess-Newsome J (1999). Pedagogical content knowledge: An introduction and orientation. In Examining pedagogical content knowledge.

[CR24] Ghavifekr S, Rosdy WAW (2015). Teaching and learning with technology: Effectiveness of ICT integration in schools. emphInternational Journal of Research in Education and Science.

[CR25] Giesbers B, Rienties B, Tempelaar D, Gijselaers W (2013). Investigating the relations between motivation, tool use, participation, and performance in an e-learning course using web-videoconferencing. Computers in Human Behavior.

[CR26] Goetz, T., Frenzel, A. C., Pekrun, R., & Hall, N. (2005). *Emotional intelligence in the context of learning and achievement* (pp. 233–253). Emotional intelligence: An international handbook.

[CR27] Harandi SR (2015). Effects of e-learning on students motivation. Procedia-Social and Behavioral Sciences.

[CR28] Heid MK (2005). Technology in mathematics education: Tapping into visions of the future. Technology-Supported Mathematics Learning Environments.

[CR29] Hidalgo, F. J. P., & Abril, C. A. H. (2020). *MOOCs: Origins, Concept and Didactic Applications: A Systematic Review of the Literature (2012–2019)* (pp. 1–27). Knowledge and Learning: Technology.

[CR30] Jacobsen DY (2019). Dropping out or dropping in? A connectivist approach to understanding participants strategies in an e-learning MOOC pilot. Technology, Knowledge and Learning.

[CR31] Jung Y, Lee J (2018). Learning engagement and persistence in massive open online courses (MOOCS). Computers and Education.

[CR32] Kaput JJ, Thompson PW (1994). Technology in mathematics education research: The first 25 years in the JRME. Journal for Research in Mathematics Education.

[CR33] Kearsley G, Shneiderman B (1998). Engagement theory: A framework for technology-based teaching and learning. Educational technology.

[CR34] Kim C, Park SW, Cozart J, Lee H (2015). From motivation to engagement: The role of effort regulation of virtual high school students in mathematics courses. Journal of Educational Technology and Society.

[CR35] Kokar MM, Endsley MR (2012). Situation awareness and cognitive modeling. IEEE Intelligent Systems.

[CR36] Kong QP, Wong NY, Lam CC (2003). Student engagement in mathematics: Development of instrument and validation of construct. Mathematics Education Research Journal.

[CR37] Kosko B (1986). Fuzzy cognitive maps. International Journal of Man-Machine Studies.

[CR38] Kuh GD (2003). What were learning about student engagement from NSSE: Benchmarks for effective educational practices. Change: The Magazine of Higher Learning.

[CR39] Lawson MA, Lawson HA (2013). New conceptual frameworks for student engagement research, policy, and practice. Review of Educational Research.

[CR40] Lepore, M., & Petruzziello, A. (2021). A situation-aware DSS to support assisted reproductive technology outcome prediction. In: *Proceedings of the 2021 IEEE Conference on Cognitive and Computational Aspects of Situation Management (CogSIMA)* (pp. 103–107). IEEE.

[CR41] Lepper MR (1988). Motivational considerations in the study of instruction. Cognition and Instruction.

[CR42] Levy Y (2007). Comparing dropouts and persistence in e-learning courses. Computers and Education.

[CR43] Likert, R. (1932). A technique for the measurement of attitudes. *Archives of Psychology.*

[CR44] Li J, Wong SC, Yang X, Bell A (2020). Using feedback to promote student participation in online learning programs: evidence from a quasi-experimental study. Educational Technology Research and Development.

[CR45] Martin AJ (2012). Part II commentary: Motivation and engagement: Conceptual, operational, and empirical clarity. Handbook of research on student engagement.

[CR46] Masika R, Jones J (2016). Building student belonging and engagement: insights into higher education students experiences of participating and learning together. Teaching in Higher Education.

[CR47] Mishra, P., & Koehler, M. J. (2007). Technological pedagogical content knowledge (TPCK): Confronting the wicked problems of teaching with technology. In Society for Information Technology and Teacher Education International Conference (pp. 2214–2226). *Association for the Advancement of Computing in Education (AACE)*.

[CR48] Pierson ME (2001). Technology integration practice as a function of pedagogical expertise. Journal of Research on Computing in Education.

[CR49] Pintrich PR, Smith DA, Garcia T, McKeachie WJ (1993). Reliability and predictive validity of the motivated strategies for learning questionnaire (MSLQ). Educational and Psychological Measurement.

[CR50] Rabardel, P. (2005). Instrument, activité et développement du pouvoir d’agir. *Entre connaissance et organisation: l’activité collective*, 251–265.

[CR51] Rabardel, P., & Samurcay, R. (2001). From artifact to instrument-mediated learning. In: *Symposium on New challenges to research on Learning* (pp. 21–23).

[CR52] Ramesh, A., Goldwasser, D., Huang, B., Daumé III, H., & Getoor, L. (2013). Modeling learner engagement in MOOCs using probabilistic soft logic. In: *NIPS workshop on data driven education* (Vol. 21, p. 62).

[CR53] Roberts LD, Howell JA, Seaman K (2017). Give me a customizable dashboard: Personalized learning analytics dashboards in higher education. Technical Knowledge Learning.

[CR54] Sarder, B., & MD, B. (2014). Improving student engagement in online courses. In: *Proceedings of the Annual ASEE: Conference*

[CR55] Shonfeld M, Magen-Nagar N (2020). The impact of an online collaborative program on intrinsic motivation, satisfaction and attitudes towards technology. Technical Knowledge and Learning.

[CR56] Skinner BF (1935). The generic nature of the concepts of stimulus and response. Journal of General Psychology.

[CR57] Skinner E, Furrer C, Marchand G, Kindermann T (2008). Engagement and disaffection in the classroom: Part of a larger motivational dynamic?. Journal of Educational Psychology.

[CR58] Stanford-Bowers DE (2008). Persistence in online classes: A study of perceptions among community college stakeholders. Journal of Online Learning and Teaching.

[CR59] Verillon, P., & Rabardel, P. (1995). Cognition and artifacts: A contribution to the study of though in relation to instrumented activity. *European Journal of Psychology of Education,* 77–101.

[CR60] Vérillon P (2000). Revisiting piaget and vygotsky. In search of a learning model for technology education. Journal of Technology Studies.

[CR61] Wolcott, L. L. (2003). Dynamics of faculty participation in distance education: Motivations, incentives, and rewards. *Handbook of Distance Education,* 549–565.

[CR62] Xiong Y, Li H, Kornhaber ML, Suen HK, Pursel B, Goins DD (2015). Examining the relations among student motivation, engagement, and retention in a MOOC: A structural equation modeling approach. Global Education Review.

[CR63] Yin RK (1981). The case study as a serious research strategy. Knowledge.

[CR64] Yin RK (2013). Validity and generalization in future case study evaluations. Evaluation.

